# Investigating Flow State and Cardiac Pre-ejection Period During Electronic Gaming Machine Use

**DOI:** 10.3389/fpsyg.2020.00300

**Published:** 2020-02-26

**Authors:** W. Spencer Murch, Mario A. Ferrari, Brooke M. McDonald, Luke Clark

**Affiliations:** ^1^Centre for Gambling Research at UBC, Department of Psychology, The University of British Columbia, Vancouver, BC, Canada; ^2^Faculty of Medicine, The University of British Columbia, Vancouver, BC, Canada

**Keywords:** flow, immersion, heart rate, gambling, slot machine, electronic gaming machine, impedance cardiography, pre-ejection period

## Abstract

Flow activities (e.g. sports and gaming) have been associated with positive affect and prolonged engagement. In the gambling field, modern electronic gaming machines (EGMs, including modern slot machines) have drawn concern as a potentially flow-inducing activity that may be associated with gambling-related harms. Current research has heavily relied on self-reported flow, and further insights may be afforded by physiological methods. We present data from three separate experiments in which self-reported gambling flow and cardiac pre-ejection period (PEP; a measure of sympathetic nervous system arousal) were examined. Male undergraduate participants gambled on a genuine EGM in a laboratory setting for a period of at least 15 min, and completed the Flow subscale of the game experience questionnaire (GEQ). Aggregated data were analyzed using multilevel regression. Although EGM gambling was not associated with significant changes in PEP across participants, we found that self-reported flow states were associated with significant decreases in PEP during the first five minutes of EGM use. Thus, participants who experienced flow showed a greater sympathetic nervous system response to the onset of gambling. Though these effects were consistent in experiments 1 and 2, in experiment 3 the effect was inverted during the same time window. We conclude that flow during EGM gambling appears to be associated with early changes in sympathetic nervous system activity, but stress that more research is needed to characterize boundary conditions and moderating factors.

## Introduction

“Although it is possible to flow while engaged in any activity, some situations appear to be designed almost exclusively so as to provide the experience of flow.”

–Mihaly Csikszentmihalyi

Flow – the trance-like experience of extreme focus on a task or activity – is often described in the context of leisure activities such as rock climbing, chess, or art (Jackson and Marsh, 1996; Csikszentmihalyi, 2014; Stavrou et al., 2015). The experience is typically associated with increases in positive affect (Asakawa, 2004; Rogatko, 2009; Csikszentmihalyi, 2014; Murch et al., 2017). According to Flow Theory, some activities are particularly adept at eliciting and sustaining the flow experience, and gambling has been proposed as one such “flow activity” (Csikszentmihalyi, 2014, p. 140, 146). A negative implication is that businesses may offer products specifically designed to encourage flow, capitalizing on prolonged or more frequent participation by individuals who are seeking to escape from stress or low mood (Schüll, 2012; Dixon et al., 2018).

Researchers first became interested in the flow-related aspects of gambling in the 1980s. Jacobs (1986) proposed that gambling activities can provide a pleasurable, trance-like sensation that reduces gamblers’ self-awareness. He suggested that this state of absorption in gambling was akin to the clinical symptom of dissociation, although in modern accounts it appears more characteristic of non-pathological or “normative” dissociation (Butler, 2006; Thomson and Jaque, 2012). Jacobs posited that this state of absorption could contribute to gambling addictions, and that these experiences could be addictive in-and-of-themselves (Jacobs, 1986, 1988). One study aimed to directly compare Csikszentmihalyi’s and Jacobs’ constructs in samples of student athletes and problem gamblers (Wanner et al., 2006). Results showed that the problem gambler group endorsed every item on both the Flow Trait Scale, and Jacobs’ “Dissociation Questionnaire” (Jacobs, 1986; Jackson and Marsh, 1996). In an earlier analysis of data in the current study, we reported high internal consistency between items on the Dissociation Questionnaire (which includes feelings of being “in a trance,” and losing track of time; Jacobs, 1986), and the Flow subscale of the GEQ (“I felt completely absorbed,” “I forgot everything around me”; Poels and De Kort, 2007; IJsselsteijn et al., 2013; Murch and Clark, 2019), again suggesting considerable overlap between Flow Theory and Jacobs’ absorption construct (but see Murch et al., 2019).

The susceptibility of regular gamblers to experiencing gambling flow is reliably associated with symptoms of disordered gambling; the Dissociation Questionnaire has been repeatedly correlated with measures of problem gambling (Kofoed et al., 1997; Diskin and Hodgins, 1999; Wanner et al., 2006; Noseworthy and Finlay, 2009; Hopley and Nicki, 2010; Cartmill et al., 2015; Murch et al., 2017, 2019; Dixon et al., 2018). In two experiments, gamblers were asked to monitor an area off-screen at the same time as they gambled on an electronic gaming machine (EGM; including modern slot machines), providing a response when target shapes appeared off-screen (Diskin and Hodgins, 1999; Murch et al., 2017). In both studies, levels of problematic gambling were associated with reduced detection of peripheral targets while gambling. This effect is consistent with the “attentional narrowing” mechanism proposed in Flow Theory (Csikszentmihalyi, 2014, p. 139). A more granular investigation of gambling flow found that specific flow experiences may have protective (losing track of time, autotelic experiences) or aggravating (senses of concentration and control) effects on gambling harms (Trivedi and Teichert, 2017; see also Palomäki and Laakasuo, 2016).

Some forms of gambling may be especially good at eliciting flow. EGMs are disproportionately associated with problem gambling (Breen and Zimmerman, 2002; MacLaren, 2015; Binde et al., 2017; Gainsbury et al., 2019). Recent accounts of EGM gambling have argued that these devices may be designed to maximize “time on device,” conceivably via flow experiences (Schüll, 2012, p. 74). In an Australian survey of gamblers who endorsed feeling “in a trance” while gambling, 79% of respondents had been using an EGM at the time (Office for Problem Gambling, 2006). Several scholars have proposed that absorption in EGM gambling may be an effective (though ultimately maladaptive) coping strategy for those seeking to avoid symptoms of depression, anxiety, or stress (Schüll, 2012; Dixon et al., 2018, 2019).

Current research relies heavily on self-report measures of flow, which can be susceptible to disruption (e.g. by introducing a secondary task; Murch et al., 2017). Psychophysiological methods may provide alternative markers for investigating the gambling flow phenomenon more covertly. Past examinations of EGM use have suggested a role for both the sympathetic (Anderson and Brown, 1984; Griffiths, 1993; Coventry and Constable, 1999; Coventry and Hudson, 2001) and parasympathetic nervous systems (Murch et al., 2017; Murch and Clark, 2019). However, little evidence exists for a link between gambling flow and physiological measures. In two experiments, we found no significant relationships between EGM flow and respiratory sinus arrhythmia, a cardiac marker of parasympathetic nervous system tone (Murch et al., 2017; Murch and Clark, 2019).

The present study evaluated the relationship between gambling flow and sympathetic nervous system arousal, indexed by cardiac pre-ejection period (PEP). PEP is an impedance cardiography-derived metric, which approximates the interval between onset of the electrical signal that stimulates left ventricular contraction (QRS complex) and opening of the aortic valve (commencement of blood efflux from the left ventricle into the aorta). In human studies and animal models, PEP has demonstrated excellent validity as an inverse measure of sympathetic arousal (Cacioppo et al., 2007, pp. 461, 619). In human studies, PEP has been observed to decrease (indicating sympathetic arousal) in response to anger, disgust, and fear emotional induction, and increase in response to happiness, sadness, and amusement (Kreibig, 2010). PEP is also sensitive to reward anticipation and delivery: PEP decreased when participants anticipated social reward (Brinkmann and Franzen, 2017), and was linearly related to reward size in delayed-match-to-sample tasks (Richter and Gendolla, 2009; Brinkmann and Franzen, 2013).

We report data from three laboratory experiments, in which self-reported flow and PEP data were collected for an EGM gambling session that lasted at least 15 min. We first hypothesized that PEP would decrease (relative to baseline) in response to EGM gambling, indicating sympathetic nervous system arousal associated with the gambling activity. We divided the gambling sessions into 5-min blocks to test the time-course of this response, as the effects of gambling on PEP may not be uniform across a gambling session. Our second and primary hypothesis proposed that EGM-related changes in PEP would interact significantly with participants’ flow ratings.

## Materials and Methods

### Participants

**FIGURE 1:**
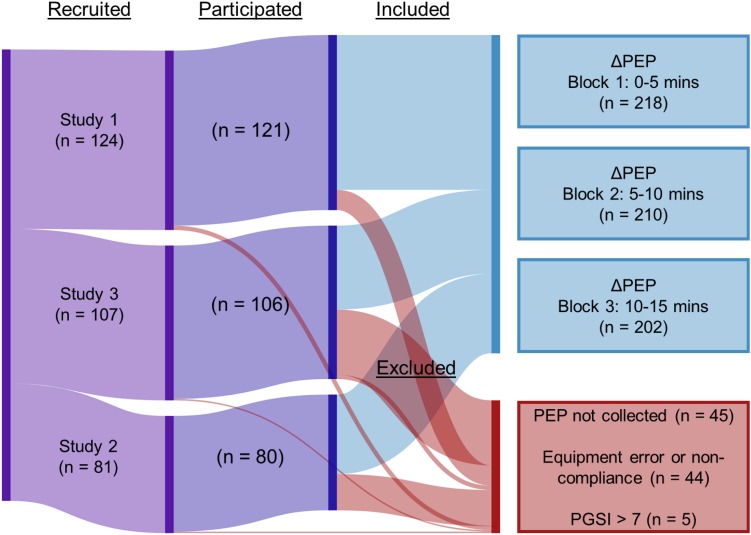
Inclusion flowchart.

The studies included in these analyses were approved by UBC’s Behavioural Research Ethics Board. Participants were recruited to three experiments conducted between 2015 and 2018 (*N*_1_ = 121, age *M* = 21.25, *SD* = 2.91; *N*_2_ = 80, age *M* = 20.55, *SD* = 2.37; *N*_3_ = 106, age *M* = 20.80, *SD* = 2.39, Figure 1). Primary analyses for Studies 1 and 2 are already published (Ferrari et al., 2018; Murch and Clark, 2019), without the measures of PEP. Study 3 has not been submitted for peer-reviewed publication (Murch, 2016). Study 1 was primarily interested in examining testosterone change in relation to EGM gambling. Study 2 looked at levels of flow and heart rate variability during EGM gambling with differing bet strategies tested within-subjects. Study 3 examined gambling immersion using a social manipulation, in which participants who provided psychophysiological data were, in some cases, tested alongside other participants seated at adjacent EGMs. Participants in Studies 2 and 3 gambled while an experimenter seated behind them monitored the physiological recording. Participants in Study 1 gambled without anyone else in the room. All participants were male undergraduate students, at least 19 years of age, who responded to an online advertisement posted by the psychology department. Most participants were compensated with partial course credit, though some participants in Study 1 were paid $15 CAD instead. Participants were included only if they were not high-risk problem gamblers (i.e. problem gambling severity index score <8, see below), had no allergies to gels or adhesives, and no current prescriptions for psychotropic or cardiac medications.

### Questionnaires

Participants completed the Problem Gambling Severity Index (PGSI), which probes past-year problem gambling symptoms (Ferris and Wynne, 2001). Responses to the nine items were rated on a 4-point Likert scale ranging from “Never” (0), to “Almost always” (3), and a total score was obtained.

After gambling on the EGMs, participants completed the Flow subscale of the GEQ (“I felt completely absorbed,” “I forgot everything around me”; Poels and De Kort, 2007; IJsselsteijn et al., 2013). In Study 2, participants completed this questionnaire after each of four 5-min gambling blocks. Responses were given on a 5-point Likert scale ranging from “Not at all” (0), to “Extremely” (4). Scores for the two items were averaged, and scores were standardized within each study. Past analysis of these items in Study 2 indicated relatively high reliability estimates (Cronbach’s α = 0.80; Murch and Clark, 2019).

### Procedure

After providing written consent, participants completed the PGSI. Individuals scoring greater than seven (indicating high risk problem gambling), on this measure were excluded from the gambling task, and instead proceeded straight to debriefing. The lab was then cleared of any additional participants and participants providing physiological data were asked to remove their shirt for electrode placement. For the impedance signal, we applied eight Ag/AgCl electrodes (Vermed, Buffalo, NY, United States); four were applied laterally on the neck and four were applied laterally on the chest below the armpit (e.g. Gramzow et al., 2008). For an electrocardiogram, we then applied three electrodes to the upper left pectoral, upper right pectoral and lower left abdomen. The data were relayed wirelessly to the RSPEC-R and NICO-R modules of a Biopac MP150 system (BIOPAC Systems, Inc., Goleta, CA, United States) recording at 1,000 Hz. Participants then put their shirts on and provided a 5-min baseline recording in a seated position. In Study 1, participants’ baseline recording was obtained at the same time as they provided a saliva sample via passive drool into a small vial. Participants in Studies 2 and 3 were instructed to close their eyes during the baseline recording, but did not provide a saliva sample.

In each study, participants gambled on a genuine EGM for at least 15 min. Each EGM was a modern, multi-line device (see Dixon et al., 2014), set on a one cent denomination (i.e. if betting on a single line, each spin would cost $0.01). In Study 1, participants gambled continuously on the EGM “Dragon’s Fire” (Scientific Games Co., Las Vegas, NV). Study 2 consisted of four 5-min gambling blocks on the EGM “Buffalo Spirit,” (Scientific Games Co., Las Vegas, NV). Study 2 participants completed the GEQ Flow questionnaire after each block. This introduced a break lasting approximately 1-min between blocks. Participants who provided PEP data in Study 3 gambled on “Double Diamond,” or “Triple Diamond,” (IGT, Las Vegas, NV, United States). Participants in all studies were provided $40–60 CAD (equivalent to 4,000–6,000 in-game credits) to use on the machine. Each study constrained participants’ betting strategies in some way. In Studies 1 and 3, a multi-line bet strategy was set, at $0.40 and $0.20, respectively, to ensure frequent reinforcement (Livingstone and Woolley, 2008; Murch et al., 2017). In Study 2, bet strategies were systematically manipulated, from one credit bet on one payline (i.e. a $0.01 bet, the minimum), to five credits bet on each of 20 paylines (i.e. $1.00 per spin). Each study involved a cash bonus incentive: participants in Study 1 were paid a $10 bonus if they finished the session in profit (i.e. over 4,000 credits), whereas participants in Studies 2 and 3 received a variable bonus from $2 to $12 based on their remaining credits.

### Processing and Analyses

Pre-ejection period is defined as the latency between Q-wave onset in an electrocardiogram, which reflects the onset of the electrical signal prompting left ventricular contraction, and the upward inflection point (B) in the derived impedance signal, dZ/dt (Cacioppo et al., 2007). In order to address our time-course hypotheses and retain comparability to baseline recordings, PEP data were partitioned into 5-min blocks. As 15 min was the shortest session length, we extracted the first three blocks (0–5, 5–10, and 10–15 min) from each study. Physiological data were visually inspected for artifacts. Blocks were excluded in cases where either the participant had run out of credit and stopped gambling, or serious artifacts precluded an accurate extraction of PEP. PEP extraction was completed using the PEP algorithm in Acqknowledge 4.4 (BIOPAC Systems, Inc., Goleta, CA, United States). Complete or partial PEP data was available for 218 participants across the three studies (Figure 1). Baseline PEP scores were subtracted from PEP scores for each block. This array of difference scores represents the change in PEP from baseline to each block, the dependent variable “ΔPEP.”

We performed three linear multilevel regression models with maximum likelihood estimation to predict ΔPEP given the block in which it was recorded, and the self-reported flow state score associated with that block. Participants in Study 2 gave flow ratings for each of the three blocks separately, while participants in Studies 1 and 3 gave a single flow rating for all blocks after the session was completed. ΔPEP blocks were nested within participants and studies, and we examined indices of model fit (AIC and BIC) to determine that these factors should be modeled as random effects (Field, 2012). Block and study number were dummy-coded. Since ΔPEP was calculated by subtracting baseline PEP levels, a value that reflects no task-related change is necessarily equal to zero, and as such the model intercept was suppressed. Models 1 and 2 directly address our hypotheses. Model 3 was included in order to explore the simple main effects of block and study on the relationship between flow and PEP. This allowed us to investigate whether any effects observed in Model 2 appeared heterogeneously across different experimental contexts.

Model 1: ΔPEP predicted by block number.

Model 2: ΔPEP predicted by block number and block-by-flow interaction terms.

Model 3: ΔPEP predicted by block number and block-by-flow-by-study interaction terms.

Analyses were performed in JASP, and R version 3.5.2, using the “nlme” package (Fox and Weisberg, 2011; R Core Team, 2018; JASP Team, 2019; Pinheiro et al., 2019). To assess the underlying assumptions of linearity and homoscedasticity, we calculated variance inflation factors and visually inspected the distributions of fitted and residual values at the levels of the factors and random effects. We were satisfied that the models did not violate the underlying assumptions of the analyses. These data and analyses have been publicly archived^1^.

## Results

The overall mean PEP during baseline blocks was 106.00 ms (*SD* = 21.79 ms). Task-related PEP levels were comparable during Block 1 (mean = 105.98 ms, *SD* = 22.17 ms), Block 2 (mean = 105.32 ms, *SD* = 19.87 ms), and Block 3 (mean = 106.47 ms, *SD* = 20.36 ms). The mean GEQ Flow score was 1.62 (*SD* = 1.12) in Study 1, 1.14 (*SD* = 0.72) in Study 2, and 1.21 (*SD* = 1.02) in Study 3, indicating mild-to-moderate levels of flow in the three experiments. A one-way ANOVA indicated that the average GEQ Flow scores differed significantly between the three studies [*F*(2,197.62) = 6.93, *p* = 0.001; Welch correction employed due to unequal variances], with higher scores in Study 1 than Study 2 (*p*_Bonferroni_ = 0.004), and Study 3 (*p*_Bonferroni_ = 0.007), but not between Studies 2 and 3 (*p*_Bonferroni_ > 0.99).

### Regression Model Results

Model 1: Overall, there was no significant change in PEP relative to baseline levels [Block 1: *B* = 0.51, *t*(408) = 0.51, *p* = 0.61; Block 2: *B* = −0.37, *t*(408) = −0.36, *p* = 0.72; Block 3: *B* = 0.61, *t*(408) = 0.60, *p* = 0.55].

Model 2: ΔPEP again did not differ significantly from baseline for Block 1 [*B* = 0.70, *t*(405) = 0.70, *p* = 0.48], Block 2 [*B* = −0.22, *t*(405) = −0.22, *p* = 0.83], or Block 3 [*B* = 0.72, *t*(405) = 0.71, *p* = 0.48]. The block-by-flow interaction term was significant for Block 1 [*B* = −1.89, *t*(405) = −2.13, *p* = 0.03], but not for Block 2 [*B* = −0.87, *t*(405) = −0.96, *p* = 0.34], or Block 3 [*B* = −0.50, *t*(405) = −0.56, *p* = 0.58]. The model fit was not significantly improved over Model 1 [χ^2^(3) = 5.24, *p* = 0.16].

**FIGURE 2:**
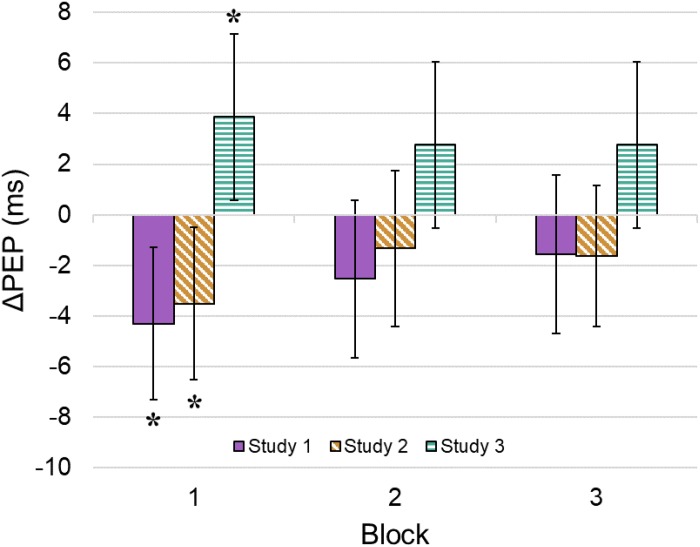
Block-by-flow-by-study interactions. Plotted values represent interaction effect coefficients summarized in Table 1 [i.e. data points represent predicted change in PEP from baseline for a participant whose immersion score was one standard deviation (SD) above the mean]. For example, the mean flow rating in Study 3 was 1.21 (*SD* = 1.02). A participant who gave a flow rating of 2.23 (+1 SD) in Study 3 is expected to have a 3.87 ms increase in PEP during Block 1 compared to their baseline level. A participant who gave a flow rating of 0.19 (–1 SD) in Study 3 is expected to have a 3.87 ms decrease in PEP during Block 1. Bars represent the 95% confidence interval. ^∗^*p* < 0.05.

Model 3: ΔPEP did not differ significantly from zero for Block 1 (*p* = 0.89, Table 1), Block 2 (*p* = 0.56), or Block 3 (*p* = 0.69). In Study 1, ΔPEP interacted significantly with flow during Block 1 (*p* = 0.01, Figure 2), but not during Block 2 (*p* = 0.11), or Block 3 (*p* = 0.33). In Study 2, ΔPEP interacted significantly with flow during Block 1 (*p* = 0.02), but not during Block 2 (*p* = 0.40), or Block 3 (*p* = 0.25). Lastly, in Study 3, ΔPEP interacted significantly with flow during Block 1 (*p* = 0.02), but not during Block 2 (*p* = 0.10), or Block 3 (*p* = 0.10). Notably, the direction of the Block 1 effect differed from those observed in Studies 1 and 2. The model fit was significantly improved over Model 2 [χ^2^(6) = 16.03, *p* = 0.01].

**TABLE 1:** Predicted ΔPEP from baseline, Model 3.

Predictor	*B*	SE (*B*)	*t*(399)	*p*
**Main effects**				
Block 1	0.14	1.00	0.14	0.89
Block 2	−0.58	1.01	−0.58	0.56
Block 3	0.40	1.01	0.40	0.69
**Interactions**				
Block 1 × Flow × Study 1	−4.29	1.54	−2.79	0.01
Block 2 × Flow × Study 1	− 2.53	1.59	−1.59	0.11
Block 3 × Flow × Study 1	−1.55	1.60	−0.97	0.33
Block 1 × Flow × Study 2	−3.51	1.54	−2.28	0.02
Block 2 × Flow × Study 2	−1.32	1.57	−0.84	0.40
Block 3 × Flow × Study 2	−1.64	1.42	−1.15	0.25
Block 1 × Flow × Study 3	3.87	1.67	2.31	0.02
Block 2 × Flow × Study 3	2.76	1.67	1.65	0.10
Block 3 × Flow × Study 3	2.77	1.67	1.65	0.10

*Flow scores have been standardized. Block and Study factors represent dummy-codes. Values in column *B* are unstandardized coefficients. Values in column SE (*B*) represent the standard error for the coefficient in that row.*

## Discussion

We tested cardiac PEP as a potential sympathetic nervous system marker of flow while undergraduate students gambled on authentic EGMs situated in a laboratory environment. We examined whether PEP changes were associated with EGM use, the stability of these levels over time, and their associations with self-reported flow, using multilevel regression models that accounted for the nested data structure. We did not observe significant change in PEP from the pre-task baseline to gambling. When we examined the interaction between task block and flow on PEP during gambling, we found that self-reported flow was associated with decreases in PEP (indicating *increased* sympathetic nervous system activity) during Block 1 (the first 5 min of gambling). When we explored this interaction within the three studies, we found opposing relationships between block and flow on ΔPEP. Studies 1 and 2 showed results consistent with Model 2: higher self-reported flow states during gambling were associated with greater decreases in PEP during Block 1 (but not Blocks 2 or 3). In Study 3, flow was associated with increased PEP (i.e. reduced sympathetic activity) and again, this effect was only statistically significant during Block 1. Taking these results together, it appears that early physiological responses to EGM use were related to increases in participants’ subsequent flow ratings. We have thus found tentative support for an association between subjective flow and fluctuations in sympathetic nervous system activity. Crucially, however, the direction of this effect may depend on particular aspects of the task procedure.

It is worth speculating on why the observed interactions with flow were limited to the first 5 min of gambling. As our flow ratings were taken at the end of the session in Studies 1 and 3, this firstly indicates that participants’ early experiences of the EGM are particularly important in accounting for variability in later flow ratings (a kind of primacy effect). These results further indicate that the initial physiological response to EGM use is an important factor in determining whether the session produces flow overall. Perhaps early experiences that produce physiological change increase the likelihood that gamblers will experience flow. In future research, it would be fruitful to take multiple flow measurements within a prolonged EGM gambling session, to characterize the subjective time-course, although such designs are challenging due to the potential for distractors to impair flow.

One possible explanation for the opposing results across the three studies is the social manipulation present in Study 3. Participants in that experiment were made aware that they may be gambling alongside other participants, and this may have impacted either their physiological response or experience of flow while gambling. Further, the gambling sessions in Study 1 (which saw the largest effect at Block 1) were conducted without an experimenter present in the room (in order to minimize any observer effects on risk-taking; Rockloff and Dyer, 2007; Rockloff et al., 2011). Thus participants’ physiological responses to the gambling task may have been moderated by these social factors, either of researchers or other participants. Alternatively, our effects could be related to participants in each study employing different betting strategies. This necessarily affected the rate of reinforcement in these studies and may have also had an impact on self-reported flow state (Murch and Clark, 2019). Consistent with past findings, we found the highest levels of flow in Study 1, which employed a 40-line bet strategy to achieve high rates of reinforcement (Livingstone and Woolley, 2008; Templeton et al., 2015). Study 3 employed a smaller, 20-line strategy, and Study 2 compared several bet strategies that varied the number of lines bet, either 1, 5, or 20. Thus, if there is a real relationship between PEP and flow during EGM use, it may depend on additional factors that we could not systematically control in this aggregated analysis.

When not accounting for flow, we observed no significant change in PEP while gambling. Previous work has typically inferred sympathetic arousal from increases in mean heart rate during gambling, including on EGMs (Anderson and Brown, 1984; Griffiths, 1993; Coventry and Norman, 1997; Coventry and Constable, 1999; Coventry and Hudson, 2001). However, the physiology of heart rate change is complex, and affected by both branches of the autonomic nervous system (Cacioppo et al., 2007). Decreases in vagal tone while gambling (Murch et al., 2017; Murch and Clark, 2019), could potentially increase heart rate while sympathetic arousal remains constant, accounting for past results. A separate possibility is that heart rate effects did reflect sympathetic arousal in past experiments, but our laboratory environment or PEP measure may have lacked the sensitivity needed to detect a sympathetic response here.

Our findings are preliminary and intended to stimulate further enquiry; they have several important limitations. First, the three study protocols differed in numerous ways, and it is possible that methodological differences drove the disparate pattern of results. Second, the laboratory environment may have attenuated physiological reactivity. EGM gambling is regarded as an appetitive psychological challenge that involves intense audiovisual stimuli, motor actions and monetary outcomes, but responses to EGM use may differ based on whether the device is situated in a gambling venue, or in a laboratory environment (c.f. Anderson and Brown, 1984). Third, participants were convenience-sampled from an undergraduate population and were not regular EGM users. This potentially diminished both physiological responses to the EGM task, and the level of flow that was reported. Fourth, participants were men, because practical application of our PEP methods precluded the recruitment of women. Fifth, the GEQ Flow scale is unidimensional, focusing on absorption states, and other measures may provide insight into different aspects of the flow state (e.g. Jackson and Eklund, 2002). Finally, the block-by-flow-by-study analytic approach was exploratory, and the available data could not clarify why opposing effects were observed between the studies. Our preliminary conclusion is that cardiac sympathetic nervous system responses early in an EGM gambling session may affect subsequent ratings of flow for that session. However, follow-up studies should be undertaken in an attempt to replicate and clarify this effect.

## Data Availability Statement

All datasets generated for this study are included in the article/supplementary material.

## Ethics Statement

The studies involving human participants were reviewed and approved by UBC Behavioural Research Ethics Board. The participants provided their written informed consent to participate in this study.

## Author Contributions

WM collected and processed the data, aggregated the data across experiments, performed the analyses, and drafted the manuscript. MF and BM collected and processed the data, and contributed to writing. LC supervised the experiments, provided the testing facilities and research funding, and contributed to writing.

## Conflict of Interest

The Centre for Gambling Research at UBC receives funding from the Province of British Columbia and the British Columbia Lottery Corporation (BCLC), a Canadian Crown Corporation. The slot machines used in the study were provided by the BCLC. The British Columbia Government and BCLC had no further involvement in the research design, methodology, conduct, analysis or write-up of the study, and impose no constraints on publishing. LC is the Director of the Centre for Gambling Research at UBC. LC has received speaker travel reimbursements/honoraria from the National Association of Gambling Studies (Australia) and the National Center for Responsible Gaming (United States), and academic consulting fees from Gambling Research Exchange Ontario (Canada) and the National Center for Responsible Gaming (United States). He has not received any further direct or indirect payments from the gambling industry or groups substantially funded by gambling. He has received royalties from Cambridge Cognition Ltd., relating to neurocognitive testing.

The remaining authors declare that the research was conducted in the absence of any commercial or financial relationships that could be construed as a potential conflict of interest.

## Footnotes

1 https://dataverse.scholarsportal.info/dataset.xhtml?persistentId=doi:10.5683/SP2/JFR1B3
